# Mining and analysis of reported ARDS population in FAERS database

**DOI:** 10.1371/journal.pone.0334500

**Published:** 2025-11-24

**Authors:** Jun Hua Lai, Yi Ping Pan, Zhi Hui Yu, Wen Fang Jang, Jun Tao Hu, Zhan Hong Tang

**Affiliations:** 1 The First Affiliated Hospital of Guangxi Medical University, Nan Ning, GuangXi, China; 2 Liuzhou Workers’ Hospital, Intensive Care Unit, The First Affiliated Hospital of Guangxi Medical University, Nan Ning, GuangXi, China; LAU Gilbert and Rose-Mary Chagoury School of Medicine: Lebanese American University School of Medicine, LEBANON

## Abstract

**Background:**

Drug-induced acute respiratory distress syndrome (ARDS) represents a severe adverse drug reaction with substantial implications for patient safety. The pathophysiology of ARDS is characterized by inflammatory cascades, endothelial dysfunction, and increased vascular permeability, culminating in pulmonary edema and compromised gas exchange. While prior studies have emphasized the impact of drug-induced adverse events (ADEs) in critical illness, comprehensive analyses leveraging large-scale databases remain underexplored.

**Methods:**

This study analyzed data from the FDA Adverse Event Reporting System (FAERS) database, covering the period from the first quarter of 2004 to the fourth quarter of 2024. The dataset underwent meticulous preprocessing, including deduplication and standardization of adverse event and drug nomenclature using the Medical Dictionary for Regulatory Activities (MedDRA) and WHO Drug Dictionaries. Disproportionality analyses were conducted using established metrics, including the Reporting Odds Ratio (ROR), Proportional Reporting Ratio (PRR), Chi-Square, Information Component (IC), and Empirical Bayes Geometric Mean (EBGM), to identify ADE signals. The final dataset comprised 18,613,992 patients, of whom 15,986 were identified as experiencing targeted ADEs.

**Results:**

The findings revealed that drug-induced ARDS is associated with significant morbidity and mortality. Among the 15,986 patients with targeted ADEs, 46.69% were male and 42.81% were female, with a median age of 55 years. Notably, 26.01% of patients were aged 65 or older. The majority of reports originated from the United States (37.86%), followed by France (13.64%) and Japan (8.65%). Severe outcomes were prevalent, with 65.04% of patients requiring hospitalization, 51.21% resulting in death, and 30.64% classified as life-threatening. Time-to-onset analysis demonstrated that 17.23% of ADEs occurred within the first 30 days of drug administration, with a median onset time of 19 days.

**Conclusion:**

This study highlights the critical nature of drug-induced ARDS and underscores the necessity for continuous pharmacovigilance and timely intervention in ADE management. The findings provide valuable insights into the demographic and clinical profiles of patients experiencing targeted ADEs, emphasizing the importance of robust drug safety surveillance. Future research should focus on elucidating risk factors, underlying mechanisms, and long-term outcomes associated with ADEs to enhance patient safety and optimize clinical management strategies.

## Introduction

The FDA Adverse Event Reporting System (FAERS) serves as a pivotal tool for post-marketing surveillance of drug and biologic safety. Utilizing the Medical Dictionary for Regulatory Activities (MedDRA) terminology, this database systematically classifies and analyzes adverse event reports submitted by healthcare professionals, manufacturers, and consumers [1,2]. FAERS plays a crucial role in detecting unexpected adverse drug reactions (ADRs) and constitutes a fundamental resource for pharmacovigilance activities. Continuously updated to reflect the evolving drug safety landscape, the database encompasses millions of reports that significantly contribute to the understanding of potential drug-associated risks [3,4].

ARDS represents a severe pulmonary condition characterized by diffuse inflammation and alveolar fluid accumulation, typically triggered by various insults including infection or trauma. The complex pathophysiology of ARDS necessitates intensive care management and mechanical ventilation support [5,6]. The disease process involves damage to the alveolar-capillary membrane, resulting in increased permeability, impaired gas exchange, and clinical manifestations of severe respiratory distress and hypoxemia [5,6]. Diagnostic criteria for ARDS include acute onset, impaired oxygenation, and characteristic radiographic findings. Notably, drug-induced ARDS has been well-documented in the literature, emphasizing the importance of vigilant monitoring for such cases within FAERS data [5,7].

Drug-induced ARDS represents a severe pulmonary complication that can be precipitated by diverse pharmacological agents, including ether, which has been documented to induce acute lung injury through its distinct pharmacokinetic properties [5]. A notable clinical case demonstrated the development of pneumonia and ARDS-like symptoms following intravenous ether administration, necessitating intensive symptomatic management [5]. Furthermore, rituximab, a monoclonal antibody utilized in the treatment of various conditions, has been associated with ARDS onset, particularly in patients with cryoglobulinemia [4]. These findings underscore the critical importance of continuous pharmacovigilance through the FAERS to promptly identify and characterize novel adverse drug reactions, especially those with potentially life-threatening consequences such as ARDS [3,8,9]. ARDS is clinically defined by the presence of acute hypoxemic respiratory failure, bilateral pulmonary infiltrates on radiographic imaging, and reduced pulmonary compliance. The etiopathogenesis of ARDS is multifactorial, encompassing infectious, traumatic, transfusion-related, and pharmacological causes. This study specifically focuses on drug-induced ARDS, which remains a diagnosis of exclusion requiring comprehensive evaluation and elimination of alternative etiological factors.

The FAERS database serves as a pivotal tool in pharmacovigilance, enabling the detection and analysis of adverse drug reactions through disproportionality analysis methods that elucidate potential associations between specific pharmacological agents and severe outcomes such as ARDS. As the largest global repository of spontaneously reported adverse drug reactions, FAERS provides invaluable insights into drug safety profiles and facilitates the identification of previously unrecognized drug-related risks [1,2,7].

## Method

### Data sources

The data utilized in this investigation were obtained from the FDA Adverse Event Reporting System (FAERS) database, which is publicly accessible through the official website of the U.S. Food and Drug Administration (FDA). The FAERS database has been available to the public since the first quarter of 2004, with quarterly updates and releases. For this study, the original ASCII data packets spanning from the first quarter of 2004 to the fourth quarter of 2024 were downloaded. These data were subsequently imported into SAS 9.4 software for data cleaning and statistical analysis. The FAERS database can be accessed at the following URL: https://fis.fda.gov/extensions/FPD-QDE-FAERS/FPD-QDE-FAERS.html.

Ethical considerations were rigorously addressed in this study. The research was conducted in compliance with established ethical standards and guidelines for secondary data analysis. Given that the data were sourced from the publicly available FAERS database, which contains de-identified information, formal ethical approval was not required. The dataset does not include any personally identifiable information and was used exclusively for research purposes. All procedures adhered to the ethical principles set forth in the Declaration of Helsinki.

### Data clearing

Given that the data in the database were self-reported, instances of duplicate entries and withdrawn/deleted reports were identified. To address this, the FDA’s official guidance document established specific protocols for data deduplication and the removal of invalid reports. In this study, data cleaning was rigorously performed in accordance with the guidelines outlined in the FDA’s official documentation. The deduplication process involved the following steps: first, duplicate reports were identified and removed based on the FDA-recommended method. This was achieved by selecting the PRIMARYID, CASEID, and FDA_DT fields from the DEMO table and sorting them by CASEID, FDA_DT, and PRIMARYID. The entry with the most recent FDA_DT value was retained, and in cases where CASEID and FDA_DT values were identical, the PRIMARYID with the highest value was preserved. Subsequently, starting from the first quarter of 2019, a quarterly deletion report list was utilized to further refine the dataset. Following deduplication, reports were removed based on the CASEID specified in the deletion report list.

### Data standardization

Adverse event nomenclature within the FAERS database was systematically encoded utilizing the Medical Dictionary for Regulatory Activities (MedDRA version 27.1), while drug nomenclature was standardized according to the World Health Organization Drug Dictionary (September 2024 release). The FAERS database employs MedDRA’s Preferred Terms (PTs) for adverse event classification, with the dictionary undergoing biannual updates in March and September. These updates necessitate recalibration of PTs and their corresponding System Organ Classes (SOCs) to maintain terminological consistency. Consequently, all PTs within the FAERS database were reclassified using the most recent MedDRA version, ensuring accurate SOC and PT assignments for subsequent analytical procedures.

For the identification of acute respiratory distress syndrome (ARDS) cases, the study implemented MedDRA’s PT coding system, specifically utilizing PT code 10001052, which precisely denotes acute respiratory distress syndrome.

### Data filtering

**Fig 1:**
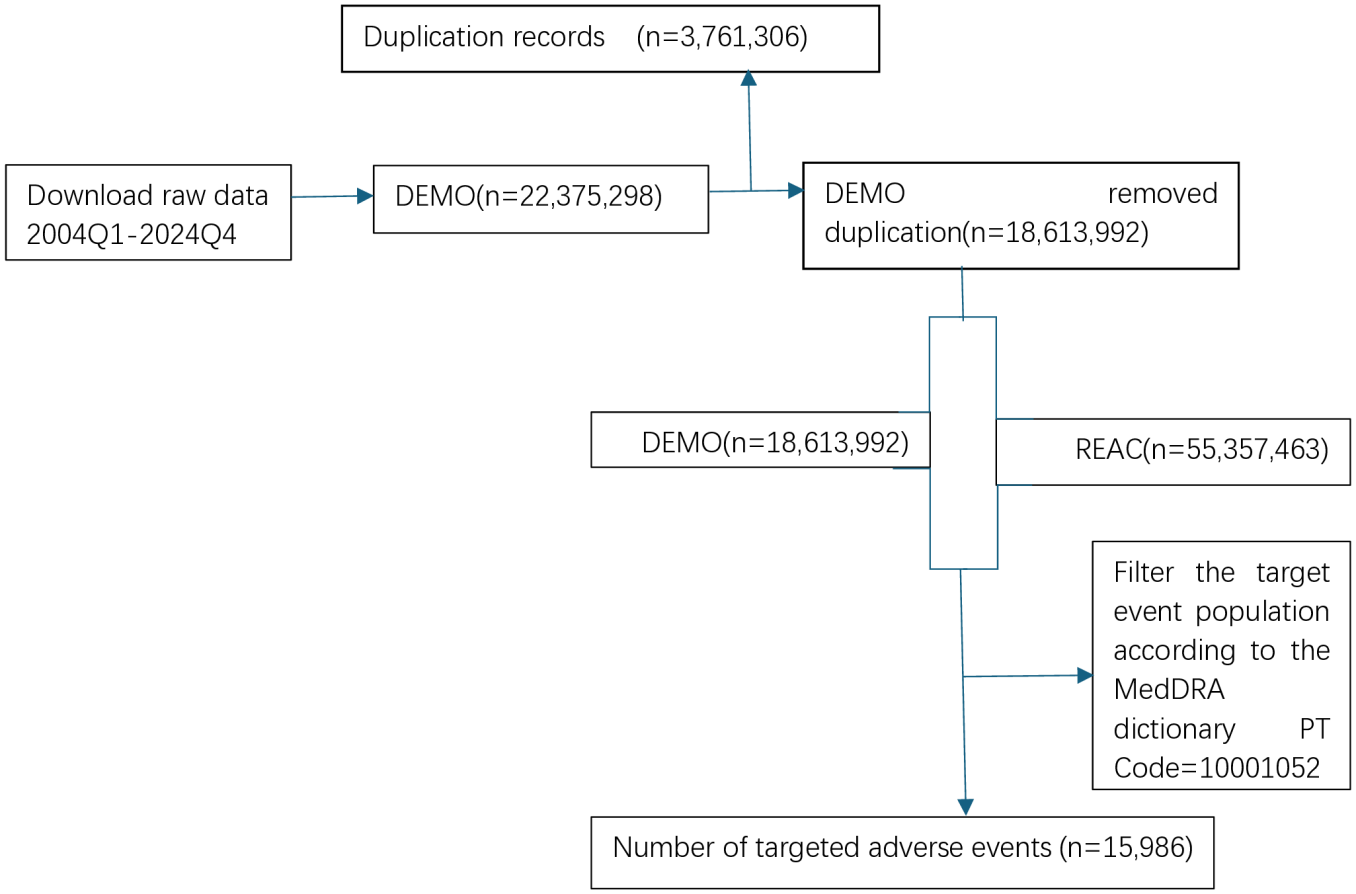
Flowchart.

In this investigation, a comprehensive dataset comprising 22,375,298 patient reports was extracted from the FAERS database. Following the application of the FDA’s weight removal reporting criteria, 3,761,306 duplicate entries from the same patients were excluded, resulting in a refined dataset of 18,613,992 unique patients. These patients collectively contributed 55,357,463 adverse event reports, with individual reports potentially encompassing multiple adverse events. Subsequent data processing yielded a final analytical cohort of 55,357,463 reports from 18,613,992 patients. Target adverse drug events (ADEs) were identified through systematic screening using preferred terms from the MedDRA dictionary (specific terms are detailed in the accompanying table). This process identified 15,986 patients exhibiting target ADEs, corresponding to 15,986 distinct target ADE reports (Fig 1).

### Data analysis

#### Other.

Calculation definition of the disproportionality approach Bayesian information component


$$\begin{array}{c} \text{IC}={\text{log}}_{\text{2}}\left(\frac{{\text{N}}_{\text{observed}}+\;\text{0}.\text{5}}{{\text{N}}_{\text{expected}}+\;\text{0}.\text{5}}\right) \end{array}$$ (1)

$$\begin{array}{c} {\text{N}}_{\text{expected}}=\frac{\left({\text{N}}_{\text{drug}}*{\text{N}}_{\text{effect}}\right)}{{\text{N}}_{\text{total}}} \end{array}$$ (2)

$$\begin{array}{c} {\text{IC}}_{\text{025}}={\text{log}}_{\text{2}}\left(\frac{{\text{N}}_{\text{observed}}+\;\text{0}.\text{5}}{{\text{N}}_{\text{expected}}+\;\text{0}.\text{5}}\right)-\text{3}.\text{3}*{\left({\text{N}}_{\text{observed}}+\text{0}.\text{5}\right)}^{-\frac{\text{1}}{\text{2}}}-\text{2}*{\left({\text{N}}_{\text{observed}}+\text{0}.\text{5}\right)}^{-\frac{\text{3}}{\text{2}}} \end{array}$$ (3)

$$\begin{array}{c} {\text{IC}}_{\text{975}}={\text{log}}_{\text{2}}\left(\frac{{\text{N}}_{\text{observed}}+\;\text{0}.\text{5}}{{\text{N}}_{\text{expected}}+\;\text{0}.\text{5}}\right)+\text{2}.\text{4}*{\left({\text{N}}_{\text{observed}}+\text{0}.\text{5}\right)}^{-\frac{\text{1}}{\text{2}}}-\text{0}.\text{5}*{\left({\text{N}}_{\text{observed}}+\text{0}.\text{5}\right)}^{-\frac{\text{3}}{\text{2}}} \end{array}$$ (4)

Nexpected: the number of case reports expected for the drug-ADR pairs.

Nobserved: the actual number of case reports for the drug-ADR pairs.

Neffect: the number of case reports for the ADR, regardless of the drug.

Ntotal: the total number of case reports in the database.

Ndrug: the number of case reports for the drug, regardless of the ADR.

1) ROR 95% CI lower bound > 1: This criterion signifies a disproportionately elevated reporting frequency relative to the background, consistent with the signal detection recommendations issued by the European Medicines Agency (EMA).
2) PRR ≥ 2 and χ² ≥ 4: These thresholds ensure that both the magnitude (a minimum two-fold increase) and the statistical significance of the association are satisfied.
3) IC-2SD > 0 (BCPNN): This Bayesian criterion requires the lower bound of the Information Component to exceed zero, thereby excluding unstable signals resulting from sparse data.
4) EBGM₀₅ > 2 (MGPS): This condition mandates that the fifth percentile of the Empirical Bayes Geometric Mean surpasses 2, effectively controlling for multiplicity and random fluctuations.

Reference: https://journals.sagepub.com/doi/10.1177/0962280211403604

Table 1 and Table 2 present an overview of commonly employed disproportionality analysis methods and their respective evaluation criteria for detecting drug adverse reaction signals. The data are structured using a 2 × 2 contingency table (Table 1) to quantify the reporting association between a specific drug and a particular adverse event. Table 2 delineates four principal disproportionality metrics: the Reporting Odds Ratio (ROR), Proportional Reporting Ratio (PRR), χ² test, Information Component (IC), and Empirical Bayes Geometric Mean (EBGM), accompanied by their computational formulas and signal detection thresholds.

**Table 1:** Two-by-two contingency table for disproportionality analysis.

Item	Target adverse events reported	Other adverse events reported	Total
Target drugs	a	b	a + b
Other drugs	c	d	c + d
Total	a + c	b + d	a + b + c + d

**Table 2:** The principles of disproportionate measurement and the criteria for signal detection.

Method	Calculation formula	Criteria
ROR	$\text{ROR}=\frac{\text{a}\;/\;\text{c}}{b\;/\;d}$	a ≥ 3 95%CI (lower limit) > 1
	$SE(lnROR)=\sqrt{\frac{\text{1}}{\text{a}}+\frac{\text{1}}{\text{b}}+\frac{\text{1}}{\text{c}}+\frac{\text{1}}{\text{d}}}$	
	$\text{9}\text{5}\%CI=\;{\text{e}}^{\text{ln}(ROR)\pm \text{1}.\text{9}\text{6}se}$	
PRR	$\text{PRR}=\frac{\text{a}\;/\;(\text{a}+\text{b})}{c\;/\;(c+d)}$	a ≥ 3 95%CI (lower limit) > 1
	$SE(lnPRR)=\sqrt{\frac{\text{1}}{\text{a}}-\frac{\text{1}}{\text{a}+\text{b}}+\frac{\text{1}}{\text{c}}-\frac{\text{1}}{\text{c}+\text{d}}}$	
	$\text{9}\text{5}\%CI=\;{\text{e}}^{\text{ln}(PRR)\pm \text{1}.\text{9}\text{6}se}$	
	$\chi 2\;=\frac{\;{\left(\text{ad}-\text{bc}\right)}^{\text{2}}(\text{a}+\text{b}+\text{c}+\text{d})}{\left(\;\text{a}+\text{b})(\text{a}+\text{c})(\text{c}+\text{d})(\text{b}+\text{d}\right)}$	a ≥ 3 PRR ≥ 2 χ2≥4
BCPNN	$\text{IC}={log}_{\text{2}}\frac{p(x,y)}{p(x)p(y)}={log}_{\text{2}}\frac{a(a+b+c+d)}{\left(a+b)(a+c\right)}$	IC025 > 0
	$\text{E}(\text{IC})={log}_{\text{2}}\frac{\left(a+\gamma \text{11})(a+b+c+d+\alpha )(a+b+c+d+\beta \right)}{\left(a+b+c+d+\gamma )(a+b+\alpha \text{1})(a+c+\beta \text{1}\right)}$	
	$V(IC)=\frac{\text{1}}{{\left(ln\text{2}\right)}^{\text{2}}}\{\left[\frac{(a+b+c+d)-a+\gamma -\gamma \text{11}}{\left(a+\gamma \text{11})(\text{1}+a+b+c+d+\gamma \right)}\right]+\left[\frac{(a+b+c+d)-(a+b)+\alpha -\alpha \text{1}}{\left(a+b+\alpha \text{1})(\text{1}+a+b+c+d+\alpha \right)}\right]+\left[\frac{(a+b+c+d)-(a+c)+\beta -\beta \text{1}}{\left(a+c+\beta \text{1})(\text{1}+a+b+c+d+\beta \right)}\right]\}$	
	$\gamma =\gamma \text{11}\frac{\left(a+b+c+d+\alpha )(a+b+c+d+\beta \right)}{\left(a+b+\alpha \text{1})(a+c+\beta \text{1}\right)}$	
	$IC-\text{2}SD=E(IC)-\text{2}\sqrt{V(IC)}$ α1=β1=1; α=β=2; γ11=1	
EBGM	$\text{EBGM}=\frac{a(a+b+c+d)}{\left(a+c)(a+b\right)}$	EBGM05 > 2
	$SE(lnEBGM)=\sqrt{\frac{\text{1}}{\text{a}}+\frac{\text{1}}{\text{b}}+\frac{\text{1}}{\text{c}}+\frac{\text{1}}{\text{d}}}$	
	$\text{9}\text{5}\%CI=\;{\text{e}}^{\text{ln}(EBGM)\pm \text{1}.\text{9}\text{6}se}$	

For ROR, a signal is identified when the observed count (a) is ≥ 3 and the lower bound of the 95% confidence interval exceeds 1. The PRR method requires a ≥ 3, PRR ≥ 2, and χ² ≥ 4 to ensure both the strength and statistical significance of the association. The Bayesian Confidence Propagation Neural Network (BCPNN) method employs the criterion IC-2SD > 0 to mitigate the influence of unstable signals arising from sparse data. The Multi-item Gamma Poisson Shrinker (MGPS) method utilizes EBGM₀₅ > 2 to account for multiple comparisons and random variability.

Furthermore, the study defines the computational approaches for the expected number of reports (Nexpected) and the observed number of reports (Nobserved), where Nexpected is calculated as the product of the total number of drug reports and the total number of adverse event reports, divided by the total number of database reports. These methodologies are grounded in extensive pharmacovigilance databases and are designed to identify disproportionately high reporting associations between drugs and adverse events, thereby uncovering potential safety signals. These approaches have been endorsed by regulatory bodies such as the European Medicines Agency (EMA) and are extensively utilized in post-marketing drug safety surveillance.

## Results

Among the 15,986 patients who developed targeted adverse drug events (ADEs), 7,464 (46.69%) were male and 6,843 (42.81%) were female. The median age of the affected patients was 55.00 years, with the 25th and 75th percentiles at 35.00 and 68.00 years, respectively. Geographically, the majority of cases were reported from the United States (6,053 patients, 37.86%), followed by France (2,180 patients, 13.64%) and Japan (1,383 patients, 8.65%). Severe outcomes were observed in 15,933 patients (99.67%), with 4,898 (30.64%) classified as life-threatening, 10,398 (65.04%) requiring hospitalization, 311 (1.95%) resulting in disability, and 8,186 (51.21%) leading to death. Additionally, 50 cases (0.31%) involved congenital malformations, and 169 cases (1.06%) necessitated intervention measures. Other serious medical events were reported in 8,337 cases (52.15%). It should be noted that the outcomes of serious reports were not mutually exclusive, as multiple options could be selected, resulting in the sum of cases exceeding the total number of serious reports (Table 3).

**Table 3:** Characteristics of AEs reports.

Indicator	Number (%)
Gender	
Female (%)	6843 (42.81)
Male (%)	7464 (46.69)
Not Specified (%)	1679 (10.50)
Age	
< 18 (%)	1326 (8.29)
18-44 (%)	3133 (19.60)
45-64 (%)	4319 (27.02)
≥ 65 (%)	4158 (26.01)
Not Specified (%)	3050 (19.08)
Age (Quantitative)	
N (Missing)	12936 (3050)
Mean (SD)	50.78 (21.94)
Median (Q1, Q3)	55.00 (35.00, 68.00)
Min, Max	0.00, 100.00
Reporting Year	
2004 (%)	479 (3.00)
2005 (%)	492 (3.08)
2006 (%)	509 (3.18)
2007 (%)	470 (2.94)
2008 (%)	489 (3.06)
2009 (%)	522 (3.27)
2010 (%)	592 (3.70)
2011 (%)	593 (3.71)
2012 (%)	618 (3.87)
2013 (%)	654 (4.09)
2014 (%)	638 (3.99)
2015 (%)	646 (4.04)
2016 (%)	681 (4.26)
2017 (%)	810 (5.07)
2018 (%)	903 (5.65)
2019 (%)	977 (6.11)
2020 (%)	1445 (9.04)
2021 (%)	1215 (7.60)
2022 (%)	1075 (6.72)
2023 (%)	1019 (6.37)
2024 (%)	1159 (7.25)
Reporter	
Consumer (%)	1345 (8.41)
Lawyer (%)	78 (0.49)
Not Specified (%)	1147 (7.18)
Other health-professional (%)	3484 (21.79)
Pharmacist (%)	3452 (21.59)
Physician (%)	6480 (40.54)
Outcome	
Life-Threatening (%)	4898 (30.64)
Hospitalization – Initial or Prolonged (%)	10398 (65.04)
Disability (%)	311 (1.95)
Death (%)	8186 (51.21)
Congenital Anomaly (%)	50 (0.31)
Required Intervention to Prevent Permanent Impairment/Damage (%)	169 (1.06)
Other (Other serious significant medical events) (%)	8337 (52.15)
Time to Onset – Days from Drug Administration (Segmented)	
0-30d (%)	2754 (17.23)
31-60d (%)	481 (3.01)
61-90d (%)	255 (1.60)
91-120d (%)	154 (0.96)
121-150d (%)	126 (0.79)
151-180d (%)	96 (0.60)
181-360d (%)	313 (1.96)
> 360d (%)	612 (3.83)
Missing or Abnormal Values (Less than 0) (%)	11195 (70.03)
Time to Onset – Days from Drug Administration (Excluding Values Less than 0)	
N (Missing)	4791 (11195)
Mean (SD)	191.28 (575.50)
Median (Q1, Q3)	19.00 (3.00, 108.00)
Min, Max	0.00, 14610.0

Note: (1) Life-threatening: events necessitating immediate medical intervention to avert mortality; (2) Disability-causing: events resulting in irreversible physical or cognitive impairment; (3) Hospitalization: events requiring in-patient medical management.

**Fig 2:**
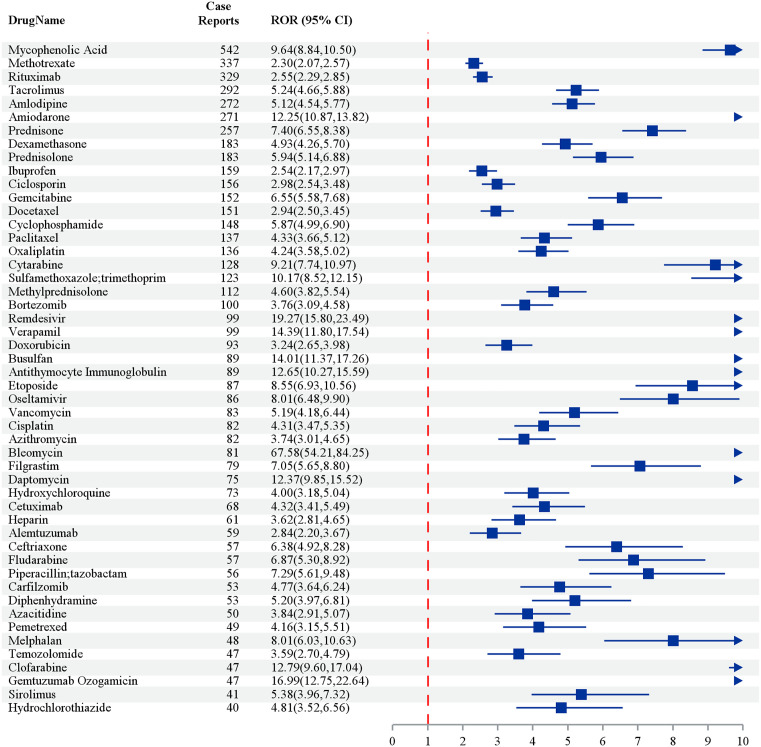
Top 50 Drugs with positive signals by frequency – by four methods (ROR PRR IC MGPS). Note: The odds ratios (OR) depicted in Fig 2 are specifically pertinent to cases of acute respiratory distress syndrome (ARDS) and should not be extrapolated to encompass all adverse events analyzed in this study.

The figure presents the relative odds ratios (RORs) and their respective 95% confidence intervals (CIs) for various drugs in relation to adverse event case reports. Mycophenolic Acid, which has the highest number of case reports (n = 542), shows a significant ROR of 9.64 (95% CI: 8.84–10.50), indicating a strong association with adverse effects. Other drugs with statistically significant associations include Methotrexate (ROR: 2.30, 95% CI: 0.27–2.57), Rituximab (ROR: 2.25, 95% CI: 2.29–2.85), and Tacrolimus (ROR: 5.24, 95% CI: 4.65–5.88). Notably, Remdesivir exhibits a significantly elevated ROR of 19.27 (95% CI: 11.80–32.49) based on 99 case reports, suggesting a potentially strong association with severe adverse effects. In contrast, Hydrochlorothiazide, with 40 case reports, shows a comparatively lower ROR of 4.81 (95% CI: 3.52–6.56) (Fig 2).

**Fig 3:**
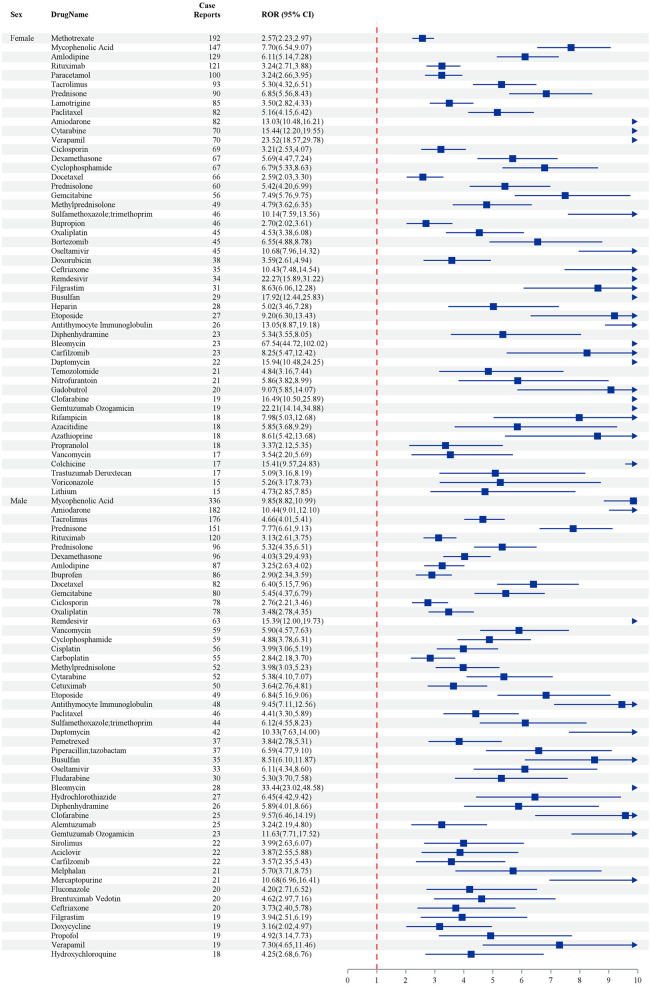
The top 50 drugs by frequency of positive signals stratified by sex – by 4 methods (ROR PRR IC MGPS). Note: The odds ratios (OR) depicted in Fig 3 are specifically pertinent to cases of acute respiratory distress syndrome (ARDS) and should not be extrapolated to encompass all adverse events analyzed in this study.

The table summarizes the relative odds ratios (ROR) for various drugs associated with adverse event reports, delineated by sex. Among female patients, Mycophenolic Acid showed the highest incidence, with 147 case reports and an ROR of 7.10 (95% CI: 5.94–8.70), indicating a significant association with adverse effects. Methotrexate also exhibited a notable association with 192 case reports and an ROR of 2.57 (95% CI: 2.32–2.97). In male patients, Mycophenolic Acid was again prominent, showing 336 reports and a markedly higher ROR of 9.83 (95% CI: 7.89–12.10). Other drugs such as Tacrolimus (male: ROR 5.73, 95% CI: 4.37–7.56) and Amiodarone (female: ROR 13.05, 95% CI: 10.18–16.21) also highlighted their risks, suggesting that targeted monitoring of these drugs is warranted due to their elevated RORs across genders (Fig 3).

**Fig 4:**
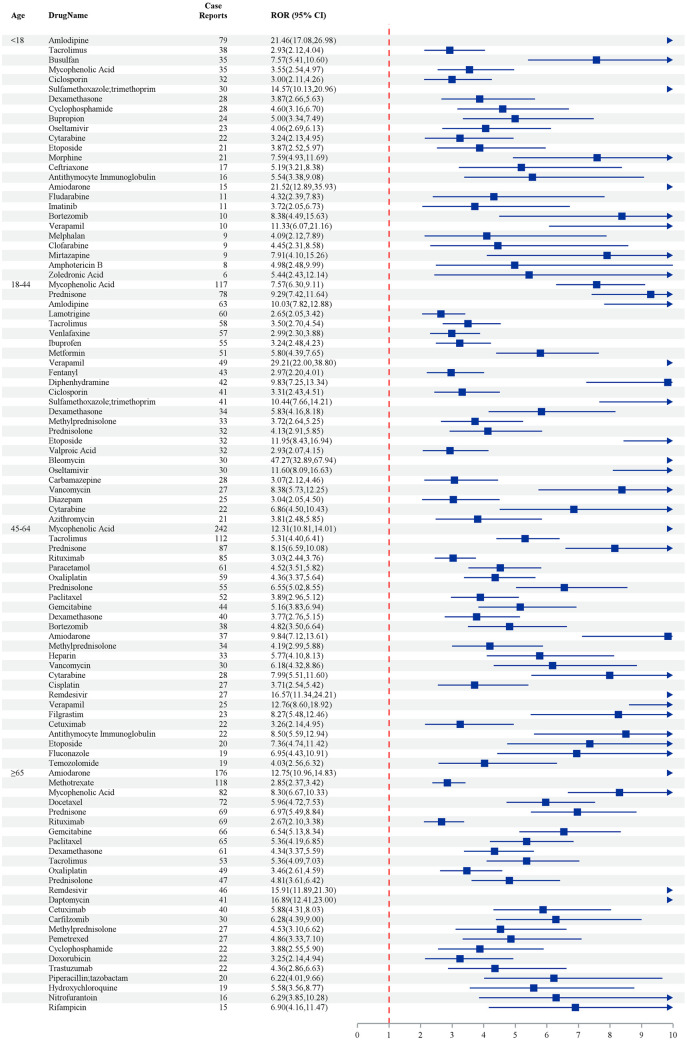
The top 25 drugs sorted by frequency of positive signals stratified by age – by 4 methods (ROR PRR IC MGPS).

Fig 4 illustrates the case reports and corresponding relative odds ratios (RORs) with 95% confidence intervals (CIs) for various drugs stratified by age groups. In the under-18 cohort, Amlodipine exhibited the highest ROR of 21.46 (95% CI: 17.08–26.98) based on 79 case reports, indicating a pronounced association with adverse events, followed by Tacrolimus with an ROR of 2.96 (95% CI: 1.12–4.04). Within the 18–44 age group, Mycophenolic Acid demonstrated the most significant association, with an ROR of 7.76 (95% CI: 4.21–11.68) derived from 117 case reports, while Amlodipine and Lamotrigine also showed notable associations (RORs: 2.70 and 2.56, respectively). In the 45–64 age category, Mycophenolic Acid remained prominent, with an ROR of 2.31 (95% CI: 1.10–4.41) based on 242 reports, alongside Tacrolimus, which exhibited an ROR of 1.31 (95% CI: 0.40–4.01). Among individuals aged 65 and older, Amlodipine was associated with 176 cases and an ROR of 1.72 (95% CI: 1.06–2.83), followed by Methotrexate, which showed an ROR of 2.82 (95% CI: 1.85–4.34) based on 52 reports. These findings collectively highlight the differential risk profiles of specific medications across distinct age demographics (Fig 4).

**Fig 5:**
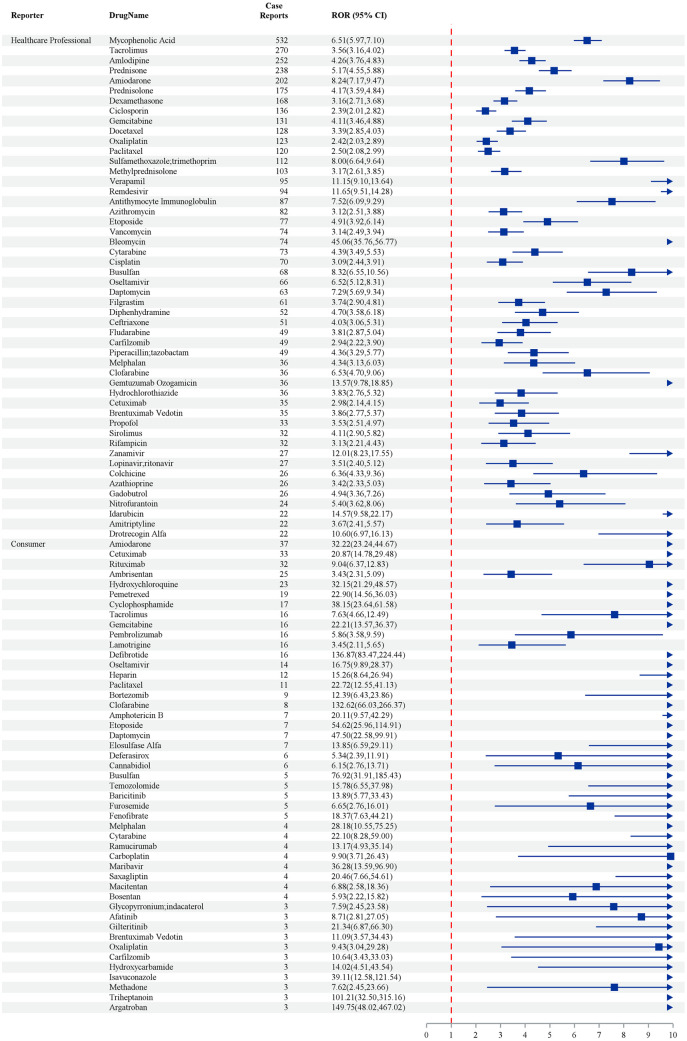
Top 50 drugs sorted by frequency of positive signals stratified by reporter – by 4 methods (ROR PRR IC MGPS).

The study presents case reports and corresponding relative odds ratios (RORs) with 95% confidence intervals (CIs) for various drugs, as reported by healthcare professionals and consumers. Mycophenolic Acid emerged as particularly notable, with 532 case reports and an ROR of 6.51 (95% CI: 5.97–7.10), indicating a statistically significant association with adverse effects. Other drugs of concern include Tacrolimus, with 270 case reports and an ROR of 3.66 (95% CI: 3.16–4.02), and Amlodipine, with 252 reports and an ROR of 2.46 (95% CI: 1.79–3.48). Among healthcare professionals, drugs such as Prednisone (ROR: 5.17, 95% CI: 4.55–5.88) and Dexamethasone (ROR: 3.16, 95% CI: 2.11–4.68) also demonstrated elevated RORs. In contrast, consumer reports highlighted Hydroxychloroquine, which exhibited an exceptionally high ROR of 32.15 (95% CI: 12.91–48.37) based on 23 cases, suggesting a substantial risk Profile (Fig 5).

**Fig 6:**
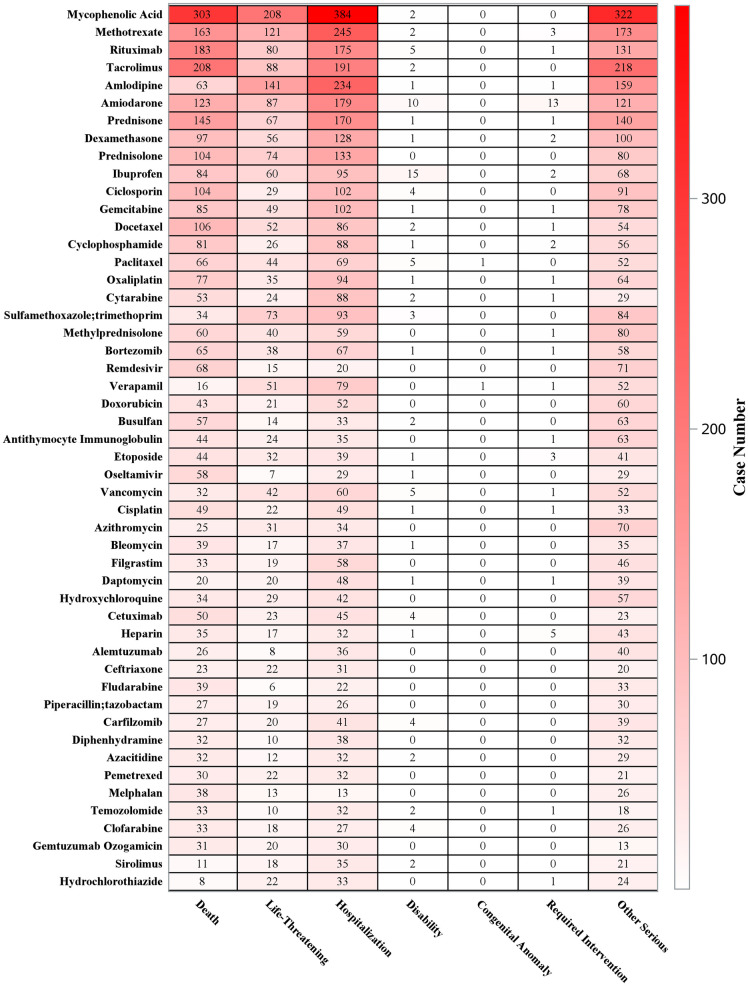
Outcomes distribution of drug patients with the top 50 positive signal frequency by 4 methods (ROR PRR IC MGPS).

The data presented delineate case frequencies and associated adverse event classifications across various pharmacological agents, with Mycophenolic Acid emerging as the most prominently reported medication, accounting for 1,386 cases. Among these, 303 cases were fatal, and 384 necessitated hospitalization. Methotrexate was documented in 721 cases, including 121 fatalities and 245 hospitalizations. Rituximab and Tacrolimus also demonstrated significant associations, with 459 and 485 cases reported, respectively. The adverse events were systematically categorized into life-threatening conditions, hospitalizations, disabilities, congenital anomalies, required interventions, and other serious outcomes, thereby facilitating the identification of critical safety concerns (Fig 6).

**Fig 7:**
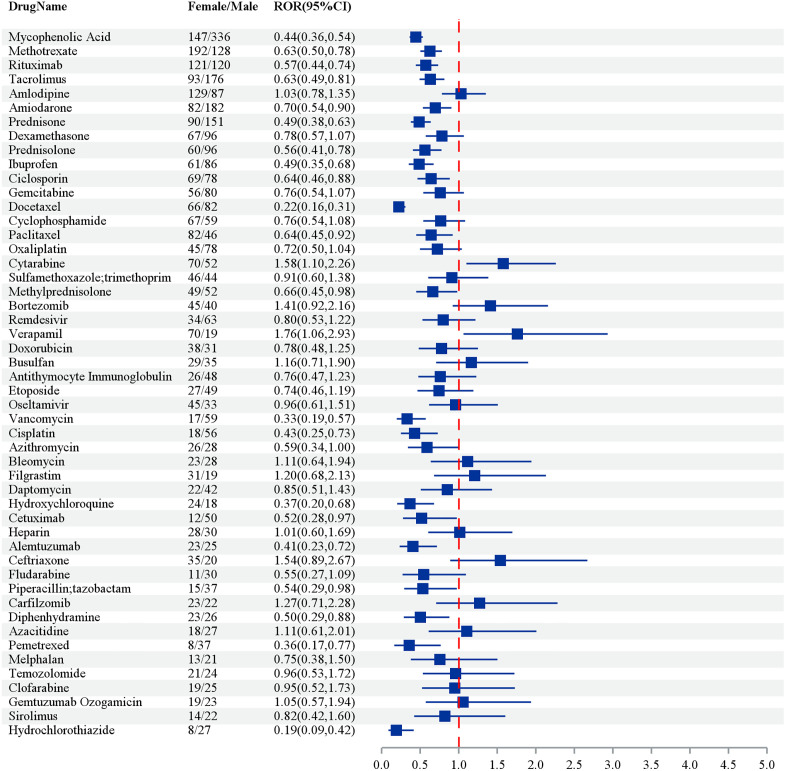
Gender Difference analysis of the top 50 drugs ranked by frequency by four methods (ROR PRR IC MGPS).

Fig 7 presents the relative odds ratios (RORs) and corresponding 95% confidence intervals (CIs) for multiple drugs, stratified by gender. Mycophenolic acid exhibits a significantly lower ROR of 0.44 (95% CI: 0.36–0.54) in females compared to males, indicating a reduced probability of adverse events in the female cohort. A comparable pattern is observed for methotrexate, with an ROR of 0.63 (95% CI: 0.50–0.78), as well as for rituximab and tacrolimus, which display RORs of 0.57 (95% CI: 0.40–0.74) and 0.63 (95% CI: 0.49–0.81), respectively, further supporting a diminished risk of adverse events in females. In contrast, amlodipine demonstrates an ROR of 1.03 (95% CI: 0.78–1.35), indicating no significant gender-based difference in risk. Notably, cytarabine and busulfan show elevated RORs of 1.58 (95% CI: 1.01–2.26) and 1.56 (95% CI: 1.06–2.29), respectively, suggesting an increased risk of adverse events among females.

Table 4 delineates the signal strength metrics for adverse drug events (ADEs) across various pharmacological agents, systematically ranked according to the volume of case reports. Each drug is characterized by its corresponding case report count, Reporting Odds Ratio (ROR) with 95% confidence interval (CI), Proportional Reporting Ratio (PRR) with 95% CI, Chi-square statistic, Information Component (IC) with IC025, and Empirical Bayes Geometric Mean (EBGM) with EBGM05. Mycophenolic Acid emerges with the highest case report count (n = 542) and a statistically significant ROR of 9.64 (95% CI: 8.84–10.50), indicative of a robust association with ADEs. Notably, agents such as Remdesivir and Bleomycin exhibit elevated ROR values exceeding 10, underscoring substantial reporting odds. In contrast, Hydrochlorothiazide demonstrates a comparatively attenuated ROR of 4.81 (95% CI: 3.52–6.56), reflecting a diminished reporting rate relative to other agents in the analysis.

**Table 4:** Signal strength of ADE at the drug level ranked by reports.

Drug NAME	Case reports	ROR(95% CI)	PRR(95% CI)	ChiSquare	IC(IC025)	EBGM(EBGM05)
Mycophenolic Acid	542	9.64 (8.84,10.50)	9.61 (8.82,10.47)	4041.35	3.22 (3.07)	9.32 (8.55)
Methotrexate	337	2.30 (2.07,2.57)	2.30 (2.07,2.57)	243.54	1.19 (1.02)	2.28 (2.04)
Rituximab	329	2.55 (2.29,2.85)	2.55 (2.29,2.85)	304.06	1.33 (1.17)	2.52 (2.26)
Tacrolimus	292	5.24 (4.66,5.88)	5.23 (4.66,5.87)	981.27	2.37 (2.17)	5.15 (4.59)
Amlodipine	272	5.12 (4.54,5.77)	5.11 (4.54,5.76)	884.76	2.33 (2.14)	5.04 (4.47)
Amiodarone	271	12.25 (10.87,13.82)	12.21 (10.83,13.77)	2743.91	3.59 (3.35)	12.02 (10.66)
Prednisone	257	7.40 (6.55,8.38)	7.39 (6.54,8.36)	1397.96	2.87 (2.65)	7.29 (6.44)
Dexamethasone	183	4.93 (4.26,5.70)	4.92 (4.25,5.69)	565.13	2.29 (2.04)	4.87 (4.21)
Prednisolone	183	5.94 (5.14,6.88)	5.93 (5.13,6.86)	742.48	2.56 (2.30)	5.88 (5.08)
Ibuprofen	159	2.54 (2.17,2.97)	2.54 (2.17,2.97)	147.24	1.34 (1.09)	2.53 (2.16)
Ciclosporin	156	2.98 (2.54,3.48)	2.97 (2.54,3.48)	202.53	1.56 (1.31)	2.96 (2.52)
Gemcitabine	152	6.55 (5.58,7.68)	6.54 (5.57,7.67)	706.12	2.70 (2.41)	6.48 (5.53)
Docetaxel	151	2.94 (2.50,3.45)	2.94 (2.50,3.45)	191.20	1.55 (1.29)	2.92 (2.49)
Cyclophosphamide	148	5.87 (4.99,6.90)	5.86 (4.98,6.89)	590.99	2.54 (2.26)	5.81 (4.94)
Paclitaxel	137	4.33 (3.66,5.12)	4.32 (3.65,5.11)	346.92	2.10 (1.82)	4.29 (3.63)
Oxaliplatin	136	4.24 (3.58,5.02)	4.23 (3.58,5.01)	332.96	2.07 (1.79)	4.20 (3.55)
Cytarabine	128	9.21 (7.74,10.97)	9.19 (7.73,10.94)	927.21	3.19 (2.85)	9.13 (7.67)
Sulfamethoxazole;trimethoprim	123	10.17 (8.52,12.15)	10.15 (8.50,12.11)	1006.73	3.33 (2.97)	10.08 (8.44)
Methylprednisolone	112	4.60 (3.82,5.54)	4.59 (3.81,5.53)	312.47	2.19 (1.87)	4.57 (3.79)
Bortezomib	100	3.76 (3.09,4.58)	3.76 (3.09,4.58)	201.47	1.90 (1.58)	3.74 (3.08)
Remdesivir	99	19.27 (15.80,23.49)	19.16 (15.74,23.34)	1694.40	4.25 (3.72)	19.05 (15.63)
Verapamil	99	14.39 (11.80,17.54)	14.33 (11.77,17.45)	1220.34	3.83 (3.36)	14.25 (11.69)
Doxorubicin	93	3.24 (2.65,3.98)	3.24 (2.64,3.98)	143.42	1.69 (1.36)	3.23 (2.63)
Busulfan	89	14.01 (11.37,17.26)	13.96 (11.34,17.18)	1064.98	3.80 (3.30)	13.89 (11.27)
Antithymocyte Immunoglobulin	89	12.65 (10.27,15.59)	12.61 (10.24,15.52)	946.17	3.65 (3.17)	12.54 (10.18)
Etoposide	87	8.55 (6.93,10.56)	8.54 (6.92,10.54)	575.85	3.09 (2.66)	8.50 (6.88)
Oseltamivir	86	8.01 (6.48,9.90)	7.99 (6.47,9.88)	523.40	2.99 (2.57)	7.95 (6.43)
Vancomycin	83	5.19 (4.18,6.44)	5.18 (4.18,6.43)	278.72	2.37 (1.98)	5.16 (4.16)
Cisplatin	82	4.31 (3.47,5.35)	4.30 (3.46,5.34)	206.88	2.10 (1.73)	4.29 (3.45)
Azithromycin	82	3.74 (3.01,4.65)	3.74 (3.01,4.65)	163.77	1.90 (1.53)	3.73 (3.00)
Bleomycin	81	67.58 (54.21,84.25)	66.31 (53.42,82.32)	5185.72	6.04 (4.88)	65.98 (52.93)
Filgrastim	79	7.05 (5.65,8.80)	7.04 (5.64,8.78)	407.30	2.81 (2.38)	7.01 (5.62)
Daptomycin	75	12.37 (9.85,15.52)	12.33 (9.83,15.46)	777.09	3.62 (3.09)	12.27 (9.78)
Hydroxychloroquine	73	4.00 (3.18,5.04)	4.00 (3.18,5.03)	163.48	1.99 (1.60)	3.99 (3.17)
Cetuximab	68	4.32 (3.41,5.49)	4.32 (3.40,5.48)	172.73	2.11 (1.69)	4.30 (3.39)
Heparin	61	3.62 (2.81,4.65)	3.62 (2.81,4.65)	115.05	1.85 (1.42)	3.61 (2.80)
Alemtuzumab	59	2.84 (2.20,3.67)	2.84 (2.20,3.66)	69.92	1.50 (1.08)	2.83 (2.19)
Ceftriaxone	57	6.38 (4.92,8.28)	6.37 (4.92,8.27)	257.43	2.67 (2.16)	6.36 (4.90)
Fludarabine	57	6.87 (5.30,8.92)	6.86 (5.29,8.90)	284.53	2.77 (2.26)	6.84 (5.27)
Piperacillin;tazobactam	56	7.29 (5.61,9.48)	7.28 (5.60,9.46)	302.43	2.86 (2.33)	7.26 (5.58)
Carfilzomib	53	4.77 (3.64,6.24)	4.76 (3.64,6.23)	157.02	2.25 (1.76)	4.75 (3.63)
Diphenhydramine	53	5.20 (3.97,6.81)	5.19 (3.96,6.80)	178.80	2.37 (1.87)	5.18 (3.95)
Azacitidine	50	3.84 (2.91,5.07)	3.84 (2.91,5.07)	104.68	1.94 (1.45)	3.83 (2.90)
Pemetrexed	49	4.16 (3.15,5.51)	4.16 (3.14,5.51)	117.35	2.05 (1.56)	4.15 (3.14)
Melphalan	48	8.01 (6.03,10.63)	7.99 (6.02,10.61)	292.82	2.99 (2.39)	7.97 (6.00)
Temozolomide	47	3.59 (2.70,4.79)	3.59 (2.70,4.78)	87.67	1.84 (1.35)	3.58 (2.69)
Clofarabine	47	12.79 (9.60,17.04)	12.75 (9.58,16.97)	507.58	3.67 (2.94)	12.72 (9.54)
Gemtuzumab Ozogamicin	47	16.99 (12.75,22.64)	16.92 (12.71,22.51)	701.97	4.08 (3.25)	16.87 (12.66)
Sirolimus	41	5.38 (3.96,7.32)	5.38 (3.96,7.30)	145.74	2.42 (1.83)	5.37 (3.95)
Hydrochlorothiazide	40	4.81 (3.52,6.56)	4.80 (3.52,6.55)	120.12	2.26 (1.68)	4.79 (3.51)
Brentuximab Vedotin	39	5.78 (4.22,7.92)	5.77 (4.22,7.90)	153.53	2.53 (1.91)	5.76 (4.21)
Defibrotide	38	16.22 (11.79,22.32)	16.15 (11.75,22.18)	538.86	4.01 (3.07)	16.11 (11.71)
Voriconazole	38	3.65 (2.66,5.02)	3.65 (2.65,5.02)	72.93	1.87 (1.31)	3.64 (2.65)
Rifampicin	36	5.33 (3.84,7.39)	5.32 (3.84,7.37)	126.01	2.41 (1.77)	5.31 (3.83)
Lopinavir;ritonavir	35	5.55 (3.98,7.74)	5.54 (3.98,7.72)	130.12	2.47 (1.82)	5.53 (3.97)
Propofol	35	5.37 (3.85,7.49)	5.37 (3.85,7.47)	124.06	2.42 (1.77)	5.36 (3.84)
Diltiazem	34	3.19 (2.28,4.47)	3.19 (2.28,4.46)	51.01	1.67 (1.10)	3.18 (2.27)
Colchicine	31	9.23 (6.49,13.14)	9.21 (6.48,13.10)	226.57	3.20 (2.36)	9.20 (6.46)
Azathioprine	30	5.05 (3.53,7.23)	5.05 (3.53,7.22)	97.22	2.33 (1.64)	5.04 (3.52)
Zanamivir	29	17.35 (12.04,25.00)	17.27 (12.01,24.84)	443.82	4.11 (2.95)	17.24 (11.97)
Vincristine	29	4.74 (3.29,6.82)	4.73 (3.29,6.81)	85.21	2.24 (1.54)	4.72 (3.28)
Gadobutrol	27	5.84 (4.01,8.53)	5.84 (4.00,8.51)	108.05	2.54 (1.77)	5.83 (3.99)
Nitrofurantoin	26	4.75 (3.23,6.99)	4.75 (3.23,6.97)	76.83	2.25 (1.50)	4.74 (3.23)
Bendamustine	26	3.18 (2.16,4.67)	3.18 (2.16,4.67)	38.76	1.67 (1.00)	3.17 (2.16)
Caspofungin	24	14.13 (9.46,21.11)	14.08 (9.44,20.99)	291.21	3.81 (2.63)	14.06 (9.41)
Gefitinib	24	3.65 (2.44,5.44)	3.64 (2.44,5.44)	45.99	1.86 (1.14)	3.64 (2.44)
Drotrecogin Alfa	24	17.52 (11.73,26.17)	17.43 (11.69,25.99)	371.33	4.12 (2.81)	17.41 (11.65)
Amitriptyline	23	4.26 (2.83,6.41)	4.25 (2.83,6.40)	57.18	2.09 (1.31)	4.25 (2.82)
Mercaptopurine	22	8.18 (5.38,12.43)	8.16 (5.37,12.39)	138.06	3.03 (2.03)	8.15 (5.36)
Idarubicin	22	22.27 (14.64,33.87)	22.13 (14.59,33.57)	443.38	4.47 (2.92)	22.10 (14.53)
Amphotericin B	22	4.79 (3.15,7.28)	4.78 (3.15,7.26)	65.74	2.26 (1.43)	4.78 (3.14)
Baricitinib	21	5.74 (3.74,8.81)	5.73 (3.74,8.79)	81.93	2.52 (1.62)	5.72 (3.73)
Micafungin	19	11.32 (7.21,17.76)	11.28 (7.20,17.68)	177.89	3.49 (2.25)	11.27 (7.18)
Daunorubicin	19	10.67 (6.80,16.74)	10.64 (6.79,16.67)	165.78	3.41 (2.20)	10.63 (6.77)
Trastuzumab Deruxtecan	19	3.44 (2.19,5.39)	3.43 (2.19,5.38)	32.73	1.78 (0.97)	3.43 (2.19)
Basiliximab	19	7.07 (4.50,11.09)	7.05 (4.50,11.06)	98.63	2.82 (1.79)	7.05 (4.49)
Decitabine	18	6.83 (4.30,10.84)	6.82 (4.30,10.82)	89.25	2.77 (1.72)	6.81 (4.29)
Ganciclovir	18	9.61 (6.05,15.27)	9.59 (6.04,15.21)	138.31	3.26 (2.06)	9.58 (6.03)
Meropenem	17	4.69 (2.91,7.55)	4.69 (2.91,7.54)	49.25	2.23 (1.28)	4.68 (2.91)
Thiotepa	16	12.51 (7.66,20.45)	12.47 (7.65,20.35)	168.74	3.64 (2.19)	12.46 (7.63)
Cilastatin;imipenem	15	4.93 (2.97,8.19)	4.93 (2.97,8.17)	46.92	2.30 (1.26)	4.92 (2.97)
Beractant	15	84.24 (50.46,140.62)	82.26 (49.88,135.67)	1203.37	6.36 (3.03)	82.19 (49.23)
Nitric Oxide	15	8.78 (5.29,14.57)	8.76 (5.28,14.52)	103.03	3.13 (1.84)	8.75 (5.27)
Hydroxyzine	15	3.87 (2.33,6.42)	3.86 (2.33,6.41)	31.83	1.95 (0.99)	3.86 (2.33)
Carmustine	14	14.52 (8.59,24.55)	14.46 (8.57,24.40)	175.33	3.85 (2.18)	14.45 (8.55)
Eptacog Alfa (Activated)	14	10.70 (6.33,18.08)	10.67 (6.32,18.00)	122.56	3.41 (1.95)	10.66 (6.31)
Ethiodized Oil	14	18.24 (10.78,30.84)	18.15 (10.76,30.60)	226.67	4.18 (2.33)	18.13 (10.72)
Valganciclovir	13	3.68 (2.14,6.34)	3.68 (2.13,6.33)	25.31	1.88 (0.85)	3.67 (2.13)
Cilgavimab;tixagevimab	13	9.98 (5.79,17.20)	9.95 (5.78,17.13)	104.62	3.31 (1.83)	9.94 (5.77)
Hydroxycarbamide	13	4.02 (2.33,6.93)	4.02 (2.33,6.92)	29.44	2.01 (0.95)	4.01 (2.33)
Dopamine	13	61.95 (35.79,107.24)	60.88 (35.51,104.37)	765.29	5.93 (2.75)	60.83 (35.15)
Norepinephrine	13	9.84 (5.71,16.96)	9.81 (5.70,16.89)	102.86	3.29 (1.82)	9.81 (5.69)
Arsenic Trioxide	12	8.57 (4.86,15.11)	8.55 (4.86,15.06)	80.02	3.10 (1.63)	8.55 (4.85)
Sevoflurane	12	4.45 (2.53,7.84)	4.45 (2.53,7.83)	32.05	2.15 (1.01)	4.44 (2.52)
Interferon Alfa-2b	12	3.84 (2.18,6.76)	3.83 (2.18,6.75)	25.13	1.94 (0.85)	3.83 (2.18)
Albumin Human	12	5.45 (3.09,9.60)	5.44 (3.09,9.58)	43.47	2.44 (1.22)	5.44 (3.09)
Foscarnet	12	14.23 (8.07,25.09)	14.18 (8.06,24.94)	146.91	3.82 (2.01)	14.17 (8.03)
Anti-D Immunoglobulin	11	5.67 (3.14,10.25)	5.66 (3.14,10.22)	42.22	2.50 (1.19)	5.66 (3.13)
Mitoxantrone	11	6.38 (3.53,11.53)	6.37 (3.53,11.50)	49.80	2.67 (1.30)	6.37 (3.52)
Tretinoin	11	5.01 (2.77,9.05)	5.00 (2.77,9.03)	35.23	2.32 (1.07)	5.00 (2.77)
Flecainide	10	3.86 (2.08,7.18)	3.86 (2.08,7.17)	21.16	1.95 (0.74)	3.86 (2.07)
Dexmedetomidine	10	5.71 (3.07,10.61)	5.70 (3.07,10.59)	38.74	2.51 (1.13)	5.70 (3.06)
Iopamidol	10	3.82 (2.05,7.10)	3.81 (2.05,7.09)	20.76	1.93 (0.73)	3.81 (2.05)
Ifosfamide	9	3.87 (2.01,7.44)	3.86 (2.01,7.43)	19.11	1.95 (0.67)	3.86 (2.01)
Vorinostat	9	5.55 (2.89,10.68)	5.55 (2.89,10.66)	33.54	2.47 (1.02)	5.54 (2.88)
Asparaginase	9	5.10 (2.65,9.81)	5.09 (2.65,9.78)	29.59	2.35 (0.94)	5.09 (2.65)
Deferoxamine	9	7.87 (4.09,15.14)	7.86 (4.09,15.09)	53.84	2.97 (1.31)	7.85 (4.08)
Ibritumomab Tiuxetan	9	8.33 (4.33,16.02)	8.31 (4.33,15.96)	57.86	3.05 (1.35)	8.31 (4.32)
Trabectedin	8	6.70 (3.35,13.41)	6.69 (3.35,13.37)	38.72	2.74 (1.07)	6.69 (3.34)
Cytarabine;daunorubicin	8	7.90 (3.95,15.81)	7.88 (3.94,15.75)	48.07	2.98 (1.20)	7.88 (3.94)
Abciximab	8	8.42 (4.21,16.86)	8.41 (4.21,16.80)	52.19	3.07 (1.24)	8.40 (4.20)
Nicardipine	8	10.10 (5.05,20.22)	10.07 (5.04,20.13)	65.38	3.33 (1.36)	10.07 (5.03)
Midostaurin	8	4.62 (2.31,9.25)	4.62 (2.31,9.23)	22.68	2.21 (0.76)	4.62 (2.31)
Sodium Polystyrene Sulfonate	8	21.17 (10.56,42.42)	21.04 (10.54,42.00)	152.71	4.39 (1.74)	21.03 (10.50)
Metyrapone	8	23.99 (11.97,48.09)	23.83 (11.94,47.55)	174.95	4.57 (1.79)	23.82 (11.88)
Ciltacabtagene Autoleucel	7	5.85 (2.78,12.27)	5.84 (2.78,12.24)	28.06	2.54 (0.84)	5.84 (2.78)
Belatacept	7	4.70 (2.24,9.86)	4.69 (2.24,9.84)	20.34	2.23 (0.66)	4.69 (2.23)
Doripenem	6	21.20 (9.50,47.32)	21.08 (9.49,46.81)	114.75	4.40 (1.35)	21.07 (9.44)
Pyrazinamide	6	17.67 (7.92,39.42)	17.58 (7.91,39.07)	93.84	4.14 (1.29)	17.58 (7.88)
Covid-19 Vaccine	6	4.72 (2.12,10.51)	4.71 (2.12,10.49)	17.55	2.24 (0.53)	4.71 (2.12)
Dactinomycin	6	7.03 (3.15,15.66)	7.02 (3.15,15.61)	30.95	2.81 (0.82)	7.01 (3.15)
Dinutuximab	6	11.04 (4.95,24.61)	11.01 (4.95,24.48)	54.59	3.46 (1.09)	11.00 (4.94)
Porfimer	6	19.26 (8.63,42.97)	19.16 (8.62,42.55)	103.24	4.26 (1.32)	19.15 (8.58)
Pentostatin	6	13.41 (6.01,29.89)	13.36 (6.01,29.69)	68.59	3.74 (1.18)	13.35 (5.99)
Plerixafor	6	11.75 (5.27,26.20)	11.72 (5.27,26.05)	58.80	3.55 (1.12)	11.71 (5.25)
Atovaquone	6	5.56 (2.49,12.38)	5.55 (2.49,12.34)	22.37	2.47 (0.66)	5.55 (2.49)
Chloroquine	6	7.71 (3.46,17.19)	7.70 (3.46,17.12)	34.96	2.94 (0.88)	7.70 (3.45)
Hydrochlorothiazide;triamterene	6	5.17 (2.32,11.52)	5.17 (2.32,11.49)	20.16	2.37 (0.60)	5.16 (2.32)
Oxygen	6	8.25 (3.70,18.38)	8.23 (3.70,18.31)	38.11	3.04 (0.92)	8.23 (3.69)
Desflurane	6	16.40 (7.35,36.58)	16.33 (7.35,36.28)	86.32	4.03 (1.26)	16.32 (7.32)
Isoflurane	6	13.54 (6.07,30.18)	13.49 (6.07,29.98)	69.36	3.75 (1.18)	13.48 (6.05)
Acetylcysteine	6	8.62 (3.87,19.21)	8.60 (3.87,19.12)	40.29	3.10 (0.95)	8.60 (3.86)
Naloxone	6	6.35 (2.85,14.15)	6.34 (2.85,14.11)	27.00	2.66 (0.75)	6.34 (2.85)
Isavuconazole	5	7.16 (2.98,17.21)	7.14 (2.98,17.15)	26.42	2.84 (0.64)	7.14 (2.97)
Nelarabine	5	7.12 (2.96,17.12)	7.10 (2.96,17.05)	26.22	2.83 (0.64)	7.10 (2.95)
Aldesleukin	5	5.14 (2.14,12.35)	5.13 (2.14,12.32)	16.62	2.36 (0.42)	5.13 (2.13)
Primaquine	5	44.74 (18.51,108.12)	44.18 (18.49,105.57)	211.02	5.46 (1.24)	44.17 (18.28)
Argatroban	5	7.76 (3.23,18.66)	7.74 (3.23,18.59)	29.36	2.95 (0.69)	7.74 (3.22)
Glycine Max;lecithin	5	14.64 (6.08,35.23)	14.58 (6.08,34.97)	63.23	3.87 (0.98)	14.57 (6.05)
Butalbital;caffeine;paracetamol	5	8.03 (3.34,19.33)	8.02 (3.34,19.25)	30.71	3.00 (0.71)	8.02 (3.33)
Barium	5	13.40 (5.57,32.26)	13.35 (5.57,32.03)	57.14	3.74 (0.94)	13.35 (5.55)
Macrogol;potassium;sodium Bicarbonate;sodium Chloride;sodium Sulfate	4	8.73 (3.27,23.30)	8.71 (3.27,23.19)	27.31	3.12 (0.48)	8.71 (3.27)
Triheptanoin	4	11.64 (4.36,31.07)	11.60 (4.36,30.87)	38.76	3.54 (0.60)	11.60 (4.35)
Peramivir	4	27.16 (10.15,72.65)	26.95 (10.15,71.56)	99.97	4.75 (0.83)	26.95 (10.07)
Nirsevimab	4	10.99 (4.12,29.34)	10.96 (4.12,29.16)	36.21	3.45 (0.58)	10.96 (4.11)
Peginterferon	4	13.49 (5.05,36.01)	13.44 (5.05,35.74)	46.05	3.75 (0.65)	13.44 (5.03)
Factor I (Fibrinogen);thrombin	4	17.92 (6.71,47.87)	17.83 (6.71,47.40)	63.55	4.16 (0.73)	17.83 (6.67)
Calcium Chloride	4	10.50 (3.93,28.02)	10.47 (3.94,27.86)	34.27	3.39 (0.56)	10.47 (3.92)
Colchicine;probenecid	4	21.24 (7.95,56.78)	21.12 (7.95,56.11)	76.67	4.40 (0.78)	21.12 (7.90)
Sulfadiazine	4	9.50 (3.56,25.35)	9.48 (3.56,25.22)	30.33	3.24 (0.52)	9.47 (3.55)
Rasburicase	4	9.09 (3.41,24.27)	9.07 (3.41,24.15)	28.73	3.18 (0.50)	9.07 (3.40)
Anidulafungin	3	9.89 (3.19,30.73)	9.87 (3.19,30.55)	23.91	3.30 (0.17)	9.87 (3.18)
Inebilizumab	3	13.49 (4.34,41.92)	13.44 (4.34,41.59)	34.55	3.75 (0.26)	13.44 (4.32)
Atenolol;chlortalidone	3	7.10 (2.29,22.06)	7.09 (2.29,21.97)	15.70	2.83 (0.05)	7.09 (2.28)
Clorazepic Acid	3	8.24 (2.65,25.58)	8.22 (2.65,25.46)	19.03	3.04 (0.11)	8.22 (2.65)
Amoxapine	3	98.00 (31.10,308.75)	95.33 (31.23,291.00)	280.04	6.57 (0.49)	95.31 (30.25)
Colistin	3	8.40 (2.71,26.10)	8.39 (2.71,25.97)	19.52	3.07 (0.11)	8.38 (2.70)
Palifermin	3	12.37 (3.98,38.42)	12.33 (3.98,38.14)	31.22	3.62 (0.24)	12.32 (3.97)
Amidotrizoic Acid	3	15.91 (5.12,49.46)	15.84 (5.12,48.99)	41.71	3.99 (0.30)	15.84 (5.09)

Note1: ranked by Reports

Note2: Signals are detected when all the following criteria are met:a ≥ 3, PRR ≥ 2 and Chi-Square ≥ 4, lower limit of 95% CI of ROR > 1, IC025 > 0, EBGM05 > 2.

### Induction time

Weber distribution was used to analyze the time of specific adverse reactions of each drug

Table 5 delineates the time-to-onset (TTO) analysis of adverse drug reactions (ADRs) for various therapeutic agents, employing the Weibull distribution test. Rituximab demonstrated a median TTO of 38 days (interquartile range [IQR]: 9.00–110.00), with a scale parameter (α) of 121.98 and a shape parameter (β) of 0.59, indicative of an early failure pattern, which aligns with the rapid manifestation of adverse events. Similarly, Docetaxel exhibited a median TTO of 11.5 days (IQR: 7.00–41.00) and an early failure profile (β = 0.83). Conversely, Oxaliplatin and Cytarabine displayed prolonged median TTOs of 44 days (IQR: 12.00–133.00) and 12 days (IQR: 5.00–27.00), respectively, characterized by random failure patterns (β = 1.00 and β = 1.03). Notably, Remdesivir exhibited the shortest median TTO of 3 days (IQR: 1.00–12.00), underscoring its propensity for rapid ADR onset (β = 0.81).

**Table 5:** Time-to-onset analysis using the Weibull distribution test.

	Cases	TTO (days)	Weibull distribution				
			Scale parameter		Shape parameter		
DrugName	n	Median(IQR)	α	95% CI	β	95% CI	Failure type
Rituximab	97	38.00 (9.00,110.00)	121.98	83.61–177.95	0.59	0.50–0.69	Early failure
Docetaxel	82	11.50 (7.00,41.00)	34.38	25.68–46.03	0.83	0.70–0.98	Early failure
Amiodarone	74	36.50 (3.00,311.00)	193.23	115.11–324.36	0.50	0.41–0.60	Early failure
Paclitaxel	73	18.00 (4.00,54.00)	46.07	32.54–65.21	0.75	0.63–0.90	Early failure
Oxaliplatin	67	44.00 (12.00,133.00)	79.33	61.03–103.13	1.00	0.81–1.22	random failure
Gemcitabine	63	55.00 (10.00,162.00)	98.21	65.89–146.38	0.67	0.56–0.81	Early failure
Remdesivir	57	3.00 (1.00,12.00)	9.29	6.44–13.40	0.81	0.66–1.00	Early failure
Bortezomib	54	11.00 (4.00,43.00)	29.58	19.18–45.61	0.68	0.55–0.84	Early failure
Methotrexate	49	249.00 (8.00,1039.00)	471.25	259.84–854.67	0.52	0.41–0.66	Early failure
Cetuximab	44	17.00 (9.00,82.00)	53.11	35.59–79.26	0.81	0.65–1.02	random failure
Daptomycin	43	18.00 (8.00,21.00)	21.75	17.06–27.72	1.34	1.06–1.68	wearout failure
Cyclophosphamide	43	28.00 (7.00,79.00)	78.31	43.03–142.54	0.54	0.44–0.67	Early failure
Antithymocyte Immunoglobulin	41	2.00 (0.00,4.00)	7.71	4.40–13.49	0.69	0.54–0.89	Early failure
Cytarabine	38	12.00 (5.00,27.00)	20.65	14.79–28.84	1.03	0.80–1.33	random failure
Gemtuzumab Ozogamicin	38	7.00 (1.00,16.00)	17.60	11.01–28.14	0.79	0.61–1.03	random failure
Tacrolimus	38	26.50 (7.00,505.00)	208.05	92.26–469.14	0.43	0.34–0.55	Early failure
Pemetrexed	37	34.00 (5.00,98.00)	53.21	33.40–84.79	0.76	0.58–1.00	Early failure
Clofarabine	34	9.00 (4.00,14.00)	16.13	10.69–24.34	0.91	0.70–1.17	random failure
Ibuprofen	34	3.00 (0.00,5.00)	5.79	4.15–8.08	1.25	0.97–1.62	random failure
Ciclosporin	31	35.00 (14.00,212.00)	162.98	85.36–311.17	0.60	0.45–0.79	Early failure
Mycophenolic Acid	30	52.00 (18.00,303.00)	215.07	100.05–462.31	0.50	0.38–0.65	Early failure
Defibrotide	30	4.00 (0.00,21.00)	34.82	17.73–68.37	0.73	0.52–1.02	random failure
Temozolomide	28	38.00 (22.00,64.50)	83.18	50.03–138.28	0.78	0.60–1.00	random failure
Azacitidine	25	19.00 (7.00,50.00)	38.45	22.18–66.63	0.79	0.58–1.08	random failure
Etoposide	24	8.50 (2.00,32.00)	24.01	14.01–41.14	0.89	0.64–1.22	random failure
Carfilzomib	23	59.00 (13.00,113.00)	78.36	50.47–121.65	1.00	0.71–1.40	random failure
Dexamethasone	23	42.00 (5.00,63.00)	80.74	43.57–149.62	0.77	0.55–1.09	random failure
Piperacillin;tazobact-am	21	2.00 (0.00,4.00)	6.74	3.90–11.65	0.98	0.67–1.44	random failure
Alemtuzumab	21	69.00 (21.00,154.00)	90.81	47.42–173.90	0.71	0.49–1.02	random failure
Oseltamivir	19	1.00 (0.00,3.00)	4.27	2.30–7.93	0.97	0.64–1.47	random failure
Busulfan	18	21.00 (16.00,70.00)	50.71	28.47–90.32	0.85	0.61–1.18	random failure
Heparin	18	6.00 (0.00,14.00)	17.51	9.30–32.98	0.95	0.62–1.44	random failure
Gadobutrol	18	0.00 (0.00,0.00)	.	. -.	.	. -.	
Doxorubicin	17	32.00 (4.00,50.00)	38.21	20.24–72.12	0.81	0.54–1.22	random failure
Gefitinib	17	65.00 (27.00,93.00)	123.09	62.44–242.63	0.77	0.55–1.08	random failure
Cisplatin	17	14.00 (11.00,23.00)	31.20	18.35–53.05	0.98	0.69–1.40	random failure
Brentuximab Vedotin	16	26.50 (7.00,76.50)	50.93	29.80–87.04	1.03	0.67–1.58	random failure
Hydroxychloroquine	16	0.00 (0.00,0.00)	138.13	2.48–7707.91	0.36	0.11–1.16	random failure
Caspofungin	15	3.00 (0.00,17.00)	20.90	6.24–69.95	0.52	0.34–0.79	Early failure
Baricitinib	15	8.00 (5.00,14.00)	25.34	9.63–66.70	0.58	0.40–0.83	Early failure
Propofol	15	0.00 (0.00,2.00)	4.43	1.60–12.29	0.84	0.48–1.47	random failure
Ceftriaxone	14	3.50 (1.00,8.00)	8.44	4.89–14.57	1.15	0.73–1.80	random failure
Bendamustine	14	75.00 (8.00,157.00)	93.31	53.50–162.74	1.07	0.65–1.74	random failure
Melphalan	14	11.00 (8.00,17.00)	18.74	12.72–27.62	1.49	1.03–2.17	wearout failure
Trastuzumab Deruxtecan	14	73.00 (8.00,214.00)	164.33	75.65–356.95	0.77	0.50–1.18	random failure
Drotrecogin Alfa	14	2.00 (2.00,5.00)	5.84	3.57–9.56	1.28	0.82–1.98	random failure
Methylprednisolone	14	8.50 (1.00,65.00)	35.72	15.04–84.83	0.69	0.44–1.09	random failure
Zanamivir	13	6.00 (2.00,13.00)	9.04	4.96–16.49	0.96	0.63–1.45	random failure
Filgrastim	13	4.00 (2.00,7.00)	8.41	4.01–17.62	0.81	0.54–1.22	random failure
Sirolimus	12	142.00 (68.50,519.00)	402.73	132.00–1228.71	0.54	0.35–0.82	Early failure
Fludarabine	11	17.00 (4.00,109.00)	59.81	19.58–182.65	0.59	0.38–0.92	Early failure
Azithromycin	11	0.00 (0.00,2.00)	4.93	1.52–16.03	0.79	0.42–1.49	random failure
Micafungin	10	1.50 (0.00,3.00)	5.19	2.58–10.47	1.21	0.67–2.20	random failure
Daunorubicin	10	17.50 (14.00,27.00)	21.34	16.30–27.94	2.42	1.47–3.99	wearout failure
Amphotericin B	10	4.50 (0.00,17.00)	21.24	8.24–54.74	0.83	0.47–1.46	random failure
Iopamidol	10	0.00 (0.00,0.00)	21.00	20.99–21.01	3998.69	3998.69–3998.69	wearout failure
Nitrofurantoin	9	17.00 (4.00,29.00)	61.81	15.84–241.13	0.54	0.33–0.90	Early failure
Anti-D Immunoglobulin	9	0.00 (0.00,0.00)	1.00	1.00–1.00	3998.69	3998.69–3998.69	wearout failure
Carmustine	9	34.00 (21.00,65.00)	57.54	27.86–118.83	0.96	0.59–1.54	random failure
Vincristine	8	23.50 (3.00,57.50)	53.43	15.39–185.47	0.63	0.36–1.11	random failure
Eptacog Alfa (Activated)	8	0.00 (0.00,1.00)	1.50	1.01–2.22	3.05	1.28–7.27	wearout failure
Ibritumomab Tiuxetan	8	24.00 (8.50,61.50)	57.55	17.02–194.64	0.61	0.37–0.99	Early failure
Meropenem	7	1.00 (0.00,5.00)	4.22	2.55–7.00	2.03	0.87–4.73	random failure
Mitoxantrone	7	13.00 (7.00,721.00)	189.97	25.09–1438.55	0.42	0.23–0.77	Early failure
Bleomycin	7	95.00 (49.00,144.00)	124.62	95.21–163.12	3.12	1.60–6.10	wearout failure
Vorinostat	7	5.00 (5.00,38.00)	28.07	7.34–107.31	0.64	0.36–1.14	random failure
Sevoflurane	7	0.00 (0.00,0.00)	.	. -.	.	. -.	
Interferon Alfa-2b	7	4.00 (0.00,24.00)	17.89	8.14–39.31	1.17	0.56–2.46	random failure
Albumin Human	7	1.00 (0.00,3.00)	2.46	1.31–4.61	1.48	0.76–2.89	random failure
Ethiodized Oil	7	2.00 (0.00,3.00)	5.53	2.58–11.86	1.37	0.66–2.82	random failure
Lopinavir;ritonavir	6	2.00 (1.00,19.00)	11.45	2.75–47.64	0.65	0.33–1.29	random failure
Cilgavimab;tixagevimab	6	10.50 (0.00,133.00)	126.84	56.85–282.96	1.47	0.54–4.00	random failure
Pentostatin	6	34.50 (28.00,330.00)	110.41	32.28–377.63	0.69	0.37–1.29	random failure
Arsenic Trioxide	6	6.50 (3.00,17.00)	10.56	5.08–21.95	1.16	0.61–2.18	random failure
Cytarabine;daunorubicin	6	5.50 (3.00,9.00)	9.06	4.39–18.69	1.18	0.65–2.11	random failure
Voriconazole	6	7.00 (4.00,24.00)	19.90	8.26–47.92	1.06	0.54–2.06	random failure
Sulfamethoxazole;trmethoprim	5	28.00 (25.00,30.00)	27.87	24.74–31.40	7.63	3.48–16.71	wearout failure
Covid-19 Vaccine	5	43.00 (34.00,124.00)	77.70	32.42–186.21	1.05	0.51–2.17	random failure
Ifosfamide	5	1.00 (0.00,2.00)	3.34	1.46–7.60	1.46	0.60–3.53	random failure
Basiliximab	5	1.00 (0.00,1.00)	6.53	1.05–40.71	0.66	0.28–1.57	random failure
Dexmedetomidine	5	0.00 (0.00,2.00)	3.36	2.20–5.12	3.46	1.09–10.99	wearout failure
Midostaurin	5	15.00 (11.00,16.00)	22.78	12.51–41.46	1.56	0.83–2.93	random failure
Prednisone	5	104.00 (36.00,138.00)	87.36	38.20–199.78	1.10	0.50–2.42	random failure
Prednisolone	5	65.00 (65.00,325.00)	185.31	48.98–701.11	0.69	0.34–1.42	random failure
Cilastatin;imipenem	4	1.00 (0.50,3.00)	2.56	1.02–6.38	1.31	0.55–3.13	random failure
Nelarabine	4	75.00 (29.00,121.00)	84.34	45.55–156.16	1.68	0.74–3.80	random failure
Decitabine	4	16.50 (11.00,27.50)	21.72	12.00–39.31	1.75	0.81–3.75	random failure
Idarubicin	4	8.50 (4.50,35.50)	16.84	4.30–65.92	0.76	0.36–1.62	random failure
Porfimer	4	3.50 (2.50,71.00)	19.10	2.75–132.47	0.54	0.26–1.11	random failure
Nitric Oxide	4	0.50 (0.00,1.00)	1.00	1.00–1.00	3998.69	3998.69–3998.69	wearout failure
Vancomycin	4	5.00 (1.00,10.00)	8.23	4.25–15.92	1.80	0.68–4.77	random failure
Trabectedin	3	1.00 (1.00,4.00)	2.24	1.02–4.93	1.53	0.64–3.64	random failure
Peginterferon	3	21.00 (21.00,186.00)	74.88	21.66–258.84	0.97	0.41–2.31	random failure
Aldesleukin	3	4.00 (4.00,4.00)	4.00	4.00–4.00	3998.69	3998.69–3998.69	wearout failure
Plerixafor	3	12.00 (0.00,55.00)	37.44	14.79–94.74	1.58	0.50–5.00	random failure
Belatacept	3	247.00 (69.00,1049.00)	456.26	138.71–1500.73	1.01	0.41–2.46	random failure
Azathioprine	3	3895.00 (37.00,3895.00)	2221.01	397.82–12399.9	0.69	0.24–1.93	random failure
Hydroxycarbamide	3	8.00 (4.00,807.00)	102.40	6.10–1719.49	0.43	0.18–1.02	random failure
Factor I (Fibrinogen);thrombin	3	1.00 (1.00,3.00)	1.90	1.02–3.54	1.93	0.81–4.59	random failure
Diltiazem	3	1.00 (0.00,12.00)	6.40	1.41–29.15	0.97	0.30–3.07	random failure
Amlodipine	3	1.00 (0.00,1759.00)	266.24	2.79–25380.9	0.32	0.10–1.02	random failure
Rifampicin	3	6.00 (1.00,18.00)	8.45	2.67–26.75	1.03	0.41–2.62	random failure
Ganciclovir	3	0.00 (0.00,19.00)	19.00	18.99–19.01	3998.69	3998.69–3998.69	wearout failure
Foscarnet	3	2.00 (0.00,9.00)	6.15	2.46–15.40	1.60	0.50–5.06	random failure
Acetylcysteine	3	1.00 (0.00,9.00)	5.17	1.35–19.73	1.09	0.34–3.47	random failure
Isavuconazole	2	38.50 (11.00,66.00)	41.97	14.07–125.17	1.34	0.42–4.25	random failure
Anidulafungin	2	2.00 (2.00,2.00)	2.00	2.00–2.00	3998.69	3998.69–3998.69	wearout failure
Valganciclovir	2	40.00 (17.00,63.00)	45.25	20.35–100.59	1.83	0.58–5.82	random failure
Dinutuximab	2	1.50 (0.00,3.00)	3.00	3.00–3.00	3998.69	3998.69–3998.69	wearout failure
CiltacabtageneAutoleucel	2	41.50 (21.00,62.00)	47.16	24.37–91.27	2.22	0.70–7.04	random failure
Inebilizumab	2	243.00 (56.00,430.00)	256.91	74.11–890.63	1.18	0.37–3.74	random failure
Tretinoin	2	2.00 (2.00,2.00)	2.00	2.00–2.00	3998.69	3998.69–3998.69	wearout failure
Abciximab	2	0.50 (0.00,1.00)	1.00	1.00–1.00	3998.69	3998.69–3998.69	wearout failure
Argatroban	2	0.00 (0.00,0.00)	.	. -.	.	. -.	
Nicardipine	2	0.50 (0.00,1.00)	1.00	1.00–1.00	3998.69	3998.69–3998.69	wearout failure
Desflurane	2	0.00 (0.00,0.00)	.	. -.	.	. -.	
Verapamil	2	0.00 (0.00,0.00)	.	. -.	.	. -.	
Beractant	2	6.00 (4.00,8.00)	6.71	4.40–10.25	3.46	1.09–10.99	wearout failure
Colistin	2	35.50 (22.00,49.00)	40.02	24.56–65.23	3.00	0.94–9.51	random failure
Deferoxamine	2	22.00 (6.00,38.00)	23.84	7.73–73.47	1.30	0.41–4.13	random failure
Rasburicase	2	0.50 (0.00,1.00)	1.00	1.00–1.00	3998.69	3998.69–3998.69	wearout failure
Palifermin	2	16.00 (0.00,32.00)	32.00	31.98–32.02	3998.69	3998.69–3998.69	wearout failure
Amidotrizoic Acid	2	0.00 (0.00,0.00)	.	. -.	.	. -.	
Triheptanoin	1	70.00 (70.00,70.00)	70.00	69.97–70.03	3998.69	3998.69–3998.69	wearout failure
Doripenem	1	1.00 (1.00,1.00)	1.00	1.00–1.00	3998.69	3998.69–3998.69	wearout failure
Peramivir	1	3.00 (3.00,3.00)	3.00	3.00–3.00	3998.69	3998.69–3998.69	wearout failure
Nirsevimab	1	23.00 (23.00,23.00)	23.00	22.99–23.01	3998.69	3998.69–3998.69	wearout failure
Dactinomycin	1	73.00 (73.00,73.00)	73.00	72.96–73.04	3998.69	3998.69–3998.69	wearout failure
Atovaquone	1	7.00 (7.00,7.00)	7.00	7.00–7.00	3998.69	3998.69–3998.69	wearout failure
Primaquine	1	120.00 (120.00,120.00)	120.00	119.94–120.06	3998.69	3998.69–3998.69	wearout failure
Chloroquine	1	3.00 (3.00,3.00)	3.00	3.00–3.00	3998.69	3998.69–3998.69	wearout failure
Flecainide	1	2.00 (2.00,2.00)	2.00	2.00–2.00	3998.69	3998.69–3998.69	wearout failure
Dopamine	1	0.00 (0.00,0.00)	.	. -.	.	. -.	
Colchicine	1	88.00 (88.00,88.00)	88.00	87.96–88.04	3998.69	3998.69–3998.69	wearout failure
Amitriptyline	1	1972.00 (1972.00,1972.00)	1972.00	1971.03–1972.97	3998.69	3998.69–3998.69	wearout failure
Diphenhydramine	1	0.00 (0.00,0.00)	.	. -.	.	. -.	
Hydroxyzine	1	2.00 (2.00,2.00)	2.00	2.00–2.00	3998.69	3998.69–3998.69	wearout failure
Naloxone	1	0.00 (0.00,0.00)	.	. -.	.	. -.	

## Discussion

This study analyzed data from the FDA Adverse Event Reporting System (FAERS) database, covering the period from Q1 2004 to Q4 2024, to investigate adverse drug events (ADEs). The dataset underwent comprehensive cleaning and standardization procedures to ensure data integrity. Duplicate reports were eliminated in accordance with FDA guidelines, while adverse event and drug names were standardized using the MedDRA Dictionary and the World Health Organization Drug Dictionary, respectively. The final dataset comprised 18,613,992 patients, of whom 15,986 with targeted ADEs were selected for detailed analysis. Demographic analysis revealed that 46.69% of patients experiencing targeted ADEs were male and 42.81% were female, with a median age of 55 years. Notably, 26.01% of patients were aged 65 or older. Geographically, the majority of reports originated from the United States (37.86%), followed by France (13.64%) and Japan (8.65%). The severity of outcomes was significant, with 65.04% of patients requiring hospitalization, 51.21% resulting in death, and 30.64% classified as life-threatening. Furthermore, 52.15% of patients experienced other serious medical events, emphasizing the critical nature of these ADEs. Time-to-onset analysis revealed that 17.23% of ADEs occurred within the first 30 days of drug administration, with a median onset time of 19 days. However, 70.03% of reports lacked or contained abnormal values for time-to-onset, potentially limiting the interpretability of these findings. Despite this limitation, the study highlights the necessity of continuous pharmacovigilance and timely intervention in managing ADEs, particularly for drugs associated with severe and life-threatening outcomes. In conclusion, this research provides critical insights into the demographic, clinical, and temporal characteristics of patients experiencing targeted ADEs.

### ARDS Pathophysiology and Drug-Induced Mechanisms

The pathogenesis of drug-induced acute respiratory distress syndrome (ARDS) is characterized by intricate pathophysiological mechanisms primarily involving alveolar-capillary barrier disruption and activation of inflammatory cascades. Central to this process is the excessive release of pro-inflammatory cytokines, particularly interleukin-6 (IL-6) and tumor necrosis factor-alpha (TNF-α), which play pivotal roles in disease progression [10,11]. This cytokine-mediated inflammatory response, often referred to as a “cytokine storm,” induces endothelial cell dysfunction and enhances vascular permeability, ultimately resulting in pulmonary edema and impaired gas exchange – hallmark features of ARDS [12].

Pharmacological agents, particularly chemotherapeutic compounds and corticosteroids, have been shown to exert direct cytotoxic effects on alveolar epithelial cells, thereby compromising the integrity of the alveolar-capillary barrier and exacerbating pulmonary injury [13,14]. Extensive research has elucidated the critical involvement of inflammatory mediators, with TNF-α demonstrating significant impact on the disruption of pulmonary tight junction proteins, leading to substantial damage to both endothelial and epithelial cellular barriers during ARDS pathogenesis [11,15]. The consequent increase in alveolar-capillary permeability represents a fundamental pathophysiological event, manifesting clinically as hypoxemia, decreased lung compliance, and diffuse alveolar damage [16,17].

### High-risk medications and signal detection

Our analysis revealed several medications exhibiting significantly elevated odds ratios (ORs) for acute respiratory distress syndrome (ARDS) development, with rituximab and mycophenolic acid demonstrating particularly strong associations. Rituximab, a monoclonal antibody targeting CD20, has been linked to pulmonary toxicity via CD20-mediated mechanisms, consistent with documented case reports of interstitial pneumonitis and acute lung injury following its administration. Similarly, mycophenolic acid, the active metabolite of mycophenolate mofetil, has been implicated in mycophenolate-induced interstitial pneumonitis, with our findings of elevated reporting odds ratios (RORs) and proportional reporting ratios (PRRs) corroborating these established associations.

The meta-analysis of bleomycin-induced lung injury models further validated our signal detection methodology, as cytotoxic agents consistently demonstrated elevated ROR and PRR values in our analysis of the FDA Adverse Event Reporting System (FAERS). This concordance between experimental models and pharmacovigilance data underscores the robustness of our findings regarding the risk of ARDS associated with chemotherapeutic agents.

Mechanistic studies have demonstrated that catecholamines, such as norepinephrine, can activate alpha-adrenergic receptors, thereby promoting lung inflammation and edema in C57BL/6 mouse sepsis models [18]. Additionally, agents like amphotericin B have been shown to induce ARDS through mechanisms involving oxidative stress and direct cytotoxicity in Sprague-Dawley rat models [19]. While these preclinical studies provide valuable mechanistic insights, their extrapolation to human clinical contexts necessitates careful consideration of species-specific differences and dosing parameters.

### Demographic observations and clinical implications

Demographic analysis indicated that 46.69% of patients experiencing targeted adverse drug events (ADEs) were male, with a median age of 55 years, and 26.01% were aged 65 years or older. Temporal analysis demonstrated that 17.23% of reported ADEs occurred within the first 30 days post-administration, with a median onset time of 19 days. These findings offer critical parameters for clinical surveillance, aiding in the identification of high-risk populations and optimal monitoring periods.

In comparison to external studies, it is important to highlight that while Shehab et al. [20] derived their incidence estimates from U.S. emergency department records predominantly involving pediatric cases, our analysis of the FAERS database encompasses a more comprehensive adult pharmacovigilance cohort. This demographic distinction necessitates cautious interpretation of direct comparisons, as pediatric and adult populations may exhibit differing susceptibility patterns and reporting behaviors.

The elevated mortality rate (51.21%) and hospitalization rate (65.04%) observed in this study are largely attributable to the inherent reporting bias of the FAERS database, which is specifically designed for pharmacovigilance of severe adverse drug reactions. The database’s focus on severe events inherently skews reported outcomes towards more critical cases, potentially inflating the observed mortality and hospitalization rates. Consequently, these findings should not be extrapolated to a broader patient population, and caution is advised when interpreting the overall risk of drug-related acute respiratory distress syndrome.

### Limitation

This study utilized data from the US Food and Drug Administration Adverse Event Reporting System (FAERS), a voluntary, spontaneous-reporting database. Given that FAERS aggregates reports from diverse sources—including patients, legal representatives, and other non-clinical entities—the diagnostic accuracy and event classification may be less reliable compared to prospectively adjudicated cohorts. Although disproportionality analysis is a well-established pharmacovigilance methodology, the clinical precision of such data is inherently constrained by reporting bias, incomplete information, and subjective interpretation by individual reporters. The lack of detailed patient-level clinical data precludes comprehensive adjustment for potential confounders. Acknowledging that patient age, sex, and ethnicity increasingly influence pharmacovigilance signal detection, we emphasize that residual demographic confounding may impact the observed associations. Future research should prioritize prospective cohort studies or randomized controlled trials to more accurately assess risk factors and elucidate the pathophysiological mechanisms underlying drug-induced acute respiratory distress syndrome.

Our analysis identified that approximately 70% of the timeline data were either missing or anomalous, presenting a substantial challenge to the validity of our timeline analysis. Although we employed multiple statistical methodologies to address these data deficiencies, the complete elimination of potential biases remains unattainable. Consequently, the interpretation of the timeline analysis results requires careful consideration, accompanied by an acknowledgment of the inherent uncertainties. The primary causes of data incompleteness and anomalies can be attributed to the following factors: (1) Incomplete reporting: As submissions to the FAERS database are voluntary, this may result in insufficiently detailed records of drug usage timelines. (2) Data entry errors: Technical errors during data entry may lead to missing or incorrect temporal information. (3) Reporting bias: An excessive focus on adverse event records may inadvertently result in the omission of comprehensive documentation of drug usage timelines. To mitigate the impact of data missingness, we implemented rigorous data cleaning procedures during the preprocessing phase, which included the systematic removal of unreasonable or erroneous records. Despite these methodological precautions, we acknowledge that data deficiencies may impose limitations on the interpretation of our research findings.

## Conclusion

This investigation highlights the critical significance of drug-induced acute respiratory distress syndrome (ARDS) and its profound implications for patient prognosis. Analysis of the FDA Adverse Event Reporting System (FAERS) database demonstrates that drug-induced ARDS is correlated with elevated hospitalization rates and mortality, underscoring the imperative for improved surveillance and intervention protocols. The findings offer critical insights into the demographic profiles of affected patients and the temporal dynamics of adverse drug events (ADEs). Notably, the study identifies specific pharmacological agents and drug classes with significant reporting odds ratios, emphasizing the necessity for targeted pharmacovigilance and further investigation into their safety profiles. Future research should prioritize elucidating the mechanistic pathways underlying these ADEs and developing preventive and therapeutic strategies for drug-induced pulmonary injury.
